# Alphataxin, a Small-Molecule Drug That Elevates Tumor-Infiltrating CD4^+^ T Cells, in Combination With Anti-PD-1 Therapy, Suppresses Murine Renal Cancer and Metastasis

**DOI:** 10.3389/fonc.2021.739080

**Published:** 2021-11-25

**Authors:** Cynthia L. Bristow, Mary Ann B. Reeves, Ronald Winston

**Affiliations:** ^1^ Alpha-1 Biologics, Long Island High Technology Incubator, Stony Brook University, Stony Brook, NY, United States; ^2^ Institute for Human Genetics and Biochemistry, Vesenaz, Switzerland; ^3^ The University of Queensland, Medicine, Brisbane, QLD, Australia

**Keywords:** alphataxin, alpha-1 antitrypsin, PD-1, elastase, cancer, CD4 T cells, immunodeficiency, chemotaxis

## Abstract

By promoting the cytotoxic function of CD8^+^ T cells, immune checkpoint inhibitor therapy, *e.g.* programmed cell death protein-1 (PD-1), effectively inhibits tumor growth in renal cell carcinoma. Yet, as many as 87% of cancer patients do not respond to immune checkpoint therapy. Importantly, cytotoxic CD8^+^ T cell function crucially relies on CD4^+^ T helper cell cytokines, in particular, tumor necrosis factor beta (TNFβ) and its CD8^+^ T cell receptor (TNFR2) in the opposing manner as immune checkpoints and their receptors. Remarkably, despite advances in immunotherapy, there are no pharmaceutical treatments that increase circulating CD4^+^ T cell counts. Nor has there been much attention given to tumor-infiltrating CD4^+^ T cells. Using data from a clinical trial (NCT01731691), we discovered that the protein alpha-1 proteinase inhibitor (α1PI, alpha-1 antitrypsin) regulates the number of circulating CD4^+^ T cells. The orally available small-molecule drug Alphataxin acts as a surrogate for α1PI in this pathway. We aimed to examine how Alphataxin affected tumor growth in a murine model of renal cell carcinoma. Alphataxin, in combination with anti-PD-1 antibody, significantly elevated the ratio of circulating and tumor-infiltrating CD4^+^ T cells. In one study, following orthotopic implantation of syngeneic renal adenocarcinoma cells, combination treatment resulted in 100% regression of tumor growth. Moreover, in mice implanted orthotopically with one log more tumor cells, doubling Alphataxin dose in combination treatment led to 100% regression in one-third of mice and 81% suppression of tumor growth in the remaining two-thirds of mice. Lung metastasis was present in monotherapy, but significantly reduced in combination-treated mice. Orally available Alphataxin, the first and only drug developed to increase CD4^+^ T cells, in combination with anti-PD-1, is a powerful therapeutic method that provides long-term remission in renal cell carcinoma and potentially other T cell-responsive cancers by increasing the number of CD4^+^ tumor-infiltrating T cells.

## Introduction

The immune checkpoint protein programmed cell death protein-1 (PD-1) expressed on CD4^+^ and CD8^+^ T cells and its ligand programmed death-ligand 1 (PD-L1) which is overexpressed by tumor cells are effective immune checkpoints that inhibit the cytotoxic function of CD8^+^ T lymphocytes (CTLs) transforming CTLs into exhausted T cells which are not well defined or possibly regulatory cells (Tregs) that suppress immune responses (1). Monoclonal antibodies that bind to PD-1 or PD-L1 are immune checkpoint inhibitors that prevent this receptor-ligand interaction and consequently allow CTLs to limit tumor growth and improve prognosis in multiple T cell-responsive cancers including renal adenocarcinoma by enhancing the cytotoxic, antitumor activity of CD8^+^ tumor infiltrating lymphocytes (TILs) (2–6). The 5-yr survival rate for patients with renal adenocarcinoma undergoing anti-PD-1 treatment is estimated to be 27.7% (7). However, despite the efficacy of checkpoint inhibitors in promoting the cytotoxic activities of CD8^+^ TILs, as many as 87% of patients do not benefit from this treatment (7, 8).

As well as immune checkpoint inhibitors to enhance cytotoxic CD8^+^ TILs activity in the tumor microenvironment, tumor control requires CD4^+^ T helper cells to provide activating cytokines to CTLs (9). In the absence of sufficient numbers of CD4^+^ T cells, specifically Type 1 helper T cells (Th1) that release cytokines IL-2, INF-λ, and TNFβ, cytotoxic CD8^+^ T cells are poorly effective (10–12). In particular, CD4^+^ T cells release tumor necrosis factor beta (TNFβ) which binds to its CD8^+^ T cell receptor (TNFR2) in the same manner, but with the opposite outcome, that tumor cells release immune checkpoints that bind to their CD8^+^ T cell receptors, e.g. PD-1 (12). Yet, there have been few investigations on the role of CD4^+^ TILs (9, 13).

We previously discovered a biochemical pathway that regulates and directs the activities of CD4^+^ T cells (14–16). This process, similar to chemotaxis, begins by the binding of plasma protein alpha-1 proteinase inhibitor (α1PI, alpha-1 antitrypsin) to cell-surface human leukocyte elastase (HLE-CS) forming a covalent-like complex that subsequently binds to members of the LDL receptor family, including very low density lipoprotein receptor (VLDLR), in a process that promotes cellular locomotion in physiological conditions (14, 15, 17–19). While many proteinase/proteinase inhibitor pairs and their fragments are known to directly or indirectly direct cellular activities, activate nuclear signaling pathways, and/or stimulate cellular locomotion, α1PI is the most abundant circulating proteinase inhibitor suggesting that it plays a prominent role in the mechanochemical process of cellular locomotion (20–23).

In two small clinical trials (NCT01370018 and NCT01731691), we previously showed that subjects infused weekly for 9 weeks with FDA-approved α1PI plasma products exhibited a dramatic, sustained increase in circulating CD4^+^ T cell numbers and/or an increase in CD4/CD8 T cell ratios in individuals infected with HIV-1 and, in parallel, in uninfected clinic patients with an α1PI genetic deficiency (Pi-ZZ) who were initiating treatment (17, 24). In contrast, there were no sustained increases in CD8^+^ T cells, monocytes, or granulocytes (24). Isolated CD4^+^ T cells from α1PI-treated subjects were stimulated and shown to be functionally competent by measuring NFκB phosphorylation and cytokine release from both Th1 (IL-2, INF-λ) and Th2 cells (IL-4, IL-10) (24). These data further revealed that α1PI interacts with immature double positive CD4^+^CD8^+^ T cells (DPs) transiting through the thymus and directs those cells to mature into CD4^+^ T cells as opposed to defaulting to CD8^+^ T cells (17, 25). Moreover, α1PI is a primary regulator of the function of mature CD4^+^ T cells by directly halting their migration within pathological tissue sites such as in bacterial infection, virus infection, host-derived inflammation, and tumors where α1PI becomes inactivated by extraneous proteinases thereby preventing cellular locomotion and transforming α1PI into a neutrophil chemoattractant, a situation creating poor cancer prognosis (15, 17, 26, 27).

Because α1PI is produced from donated human plasma, it is limited in availability and impractical for treating patients with immunodeficiencies resulting secondary to infection, inflammation, or cancer. An optimal treatment would be an orally available small molecule drug that mimics the activity of α1PI. We previously screened a panel of small molecules known to bind to granule-associated human leukocyte elastase (HLE-G) to determine whether any might also bind to HLE-CS (28). In preclinical *in vitro* studies, modification of one of those small molecules, Alphataxin, mimics α1PI activity by binding to HLE-G and HLE-CS as well as stimulating cellular locomotion and endocytosis.

Here we aimed to investigate the effects of Alphataxin on the immune system and tumors in a well-characterized T cell-responsive murine tumor model by implanting syngeneic renal tumor cells. We found that Alphataxin increased the normally circulating numbers of CD4^+^ T cells, immature DPs, and CD4/CD8 ratio. Notably, we showed that Alphataxin, as monotherapy, increased the number of CD4^+^ TILs and suppressed tumor growth, and when combined with anti-PD-1 immunotherapy, significantly suppressed or regressed tumor growth. Remarkably, metastasis was significantly reduced in the combination treatment arm. Thus, Alphataxin treatment is efficacious as a monotherapy in renal cell cancer in mice, enhances anti-PD-1 therapy, and potentially could expand the number of cancer patients who respond to checkpoint inhibitor therapy.

## Materials and Methods

### Clinical Trial NCT01731691

This previously described clinical trial was a double-blind, randomized study (17). Written informed consent was received from 12 individuals, 8 HIV-1-infected individuals and 4 uninfected controls. Blood was collected weekly at the same time of day at baseline and for 8 subsequent weeks from uninfected, untreated controls (n=4) and from HIV-1-infected individuals who were treated weekly with Prolastin-C (n=3) or with placebo (n=5). Inclusion criteria for HIV-1 infected subjects were: i) active α1PI below 11 μM; ii) one year history with CD4^+^ lymphocytes at levels ranging between 200 and 600 cells/μl; iii) absence of symptoms suggestive of HIV-1 disease progression; iv) adequate suppression of virus (<1000 HIV RNA/ml); and v) history of compliance with antiretroviral medication. Grifols Biotherapeutics contributed a sufficient quantity of Prolastin-C (lot# 26NLK52) for administration of 8 weekly infusions at a dose of 120 mg/kg. The study protocol was approved by Copernicus Group Independent Institutional Review Board, Durham, NC. Drug delivery and blood collection were performed weekly at ACRIA, New York, NY, and blood samples were shipped to ICON Central Laboratories for analysis. No adverse effects were reported by any volunteers, and all volunteers remained in the study for the full period.

### Cells and Reagents

A renal adenocarcinoma cell line, Renca, derived from a tumor that spontaneously arose in BALB/c mice, was obtained from the American Type Culture Collection (ATCC). Renca cells stably expressing green fluorescent protein (GFP) and firefly luciferase (Renca-GL) were generously provided by Dr. Thomas Griffith, University of Minnesota (29). Alphataxin, (CAS# 19379-33-0) was chemically synthesized (BOC Sciences, Shirley, NY) and delivered to mice daily by oral gavage in Dulbecco’s phosphate buffered saline (DPBS). Anti-PD-1 antibody (BioXCell, West Lebanon, NH, BE0146) was delivered to mice twice weekly *via* intraperitoneal (IP) injection.

### Animal Studies

Animals studies were conducted according to IACUC-approved protocols as detailed herein. Any mouse displaying moribundity, prolonged signs of distress (*e.g.* labored breathing, severe diarrhea), body weight loss exceeding 15% from treatment initiation, body weight or tumor volume greater than 2000 mm^3^ were euthanized. The Renca adenocarcinoma cell line spontaneously arose in female BALB/c mice and accordingly, female mice were used in the murine models of renal adenocarcinoma (30).

### Circulating T Cells in Healthy Mice

To examine the efficacy of Alphataxin to increase circulating T lymphocyte numbers, 8-wk-old male C57BL/6 mice were randomly assigned by the Division of Laboratory Animal Resources, Stony Brook University (IACUC# 250725) to one of 2 arms (6 mice/arm): vehicle control (DPBS) or Alphataxin (5 mg/kg in 10 ml/kg). Alphataxin was delivered daily by oral gavage for 5 days consecutively per week. Blood was collected retro-orbitally weekly for flow cytometry using the whole-blood staining protocol. Male mice were used because they were readily available on site.

### Subcutaneous Tumor Model

The subcutaneous model was conducted by AJES Life Sciences, Stony Brook, NY (IACUC# 764665-4) to examine the influence of Alphataxin on tumor growth. Renca cells (2x10^5^) in 100 μl Hank’s balanced salt solution (HBSS) mixed 1:1 with Matrigel (BD Biosciences) were subcutaneously implanted into the right dorsal flank of 8-wk-old female BALB/c mice. Tumor size was monitored with calipers every other day. Tumors were allowed to grow for 14 days after implantation until they had reached a volume of 45-50 mm^3^. Subsequently, Alphataxin was delivered daily by oral gavage, and anti-PD-1 antibody was delivered twice weekly by IP injection. The study included the following 8 arms with 8 randomly assigned mice/arm: no treatment, anti-PD-1 (7 mg/kg), Alphataxin (2.5 mg/kg), Alphataxin (5 mg/kg), Alphataxin (10 mg/kg), Alphataxin (2.5 mg/kg) + anti-PD-1 (7 mg/kg), Alphataxin (5 mg/kg) + anti-PD-1 (7 mg/kg), and Alphataxin (10 mg/kg) + anti-PD-1 (7 mg/kg).

### Orthotopic Tumor Models

For a study conducted by AJES Life Sciences (IACUC# 764665-4), orthotopic implantation was achieved by injecting 5 x 10^2^ Renca cells in 100 μl of HBSS mixed 1:1 with Matrigel (BD Biosciences) into the left kidney of 8-wk-old female BALB/c mice, and treatment was initiated 7 days after implantation. This study comprised the following 4 arms with 6 randomly assigned mice/arm: no treatment, Alphataxin (5 mg/kg), anti-PD-1 (7 mg/kg), and Alphataxin (5 mg/kg) + anti-PD-1 (7 mg/kg). Body weight was determined three times per week. Blood was collected weekly by retro-orbital bleed for enumeration of CD3^+^, CD8^+^, CD4^+^ T cells and DPs using the whole-blood staining protocol for flow cytometry as described in detail herein. The size of each orthotopically implanted tumor was determined at the end of the study (21 days after treatment initiation) by measuring the difference between the weight of the left tumor-bearing kidney and the right non-tumor bearing kidney. Both left and right kidneys from 2 representative mice from each of the 4 treatment arms were preserved in formalin and shipped to Global VetPathology (Montgomery Village, MD) for staining of cryosections by H&E and by immunohistochemistry to detect CD4^+^ and CD8^+^ TILs. TILs were counted in all fields of digital slides at 20x magnification using Aperio ImageScope software.

For the study, conducted by Charles River (Morrisville, NC, IACUC# 990301), orthotopic implantation was achieved by injecting 2.5 x 10^5^ Renca-GL cells or 5 x 10^3^ Renca-GL cells, respectively, in 100 μl of phosphate buffered saline (PBS) into the left kidney of 8-12-wk-old female BALB/c mice. Mice were sorted 1 day after implantation into four groups (n=6/group) according to bioluminescent flux values (BLI) to assure equivalent baseline BLI per group. After sorting, treatment was initiated comprising 4 arms with 6 mice/arm and the Alphataxin dose doubled to 10 mg/kg. Body weight was determined every other day. The size of orthotopically implanted tumors derived from Renca-GL cells was determined weekly by measuring the dorsal (kidney) and shielded ventral (lung) BLI until mice were determined to be moribund or until the endpoint of the study (67 days). Blood was collected weekly by mandibular bleed to count the numbers of CD3^+^, CD4^+^, CD8^+^ T cells, and DPs by flow cytometry using the lysed-blood staining protocol described in detail herein. Weekly blood collection and BLI imaging were not performed on the same day. At moribundity or on the last day of the study, full blood volume (0.175 ml per mouse) was collected and shipped to Antech Diagnostics (New Hyde Park, NY) to conduct a complete blood cell count with manual differential. The tumor-bearing left kidney was preserved in formalin for shipping to Pathology Associates where cryosections were stained to detect CD4^+^ TILs (rabbit anti-mouse CD4, Abcam, Cat # ab183685) and CD8^+^ TILs (rabbit anti-mouse CD8, Synaptic Systems Cat # 361003) using Indirect Envision+ System – HRP labelled polymer anti-rabbit (Dako). TILs were counted in all fields of digital slides at 20x magnification by 2 independent investigators using Aperio ImageScope software.

On day 25 of treatment, Charles River reported 2 non-treatment-related deaths. One animal in the Alphataxin treatment arm was reported to have been found dead, and necropsy revealed the animal with an abdominal cavity filled with blood and a large mass surrounding the left tumor-bearing kidney. One animal in the combination treatment arm was reported to have been found dead and beyond necropsy.

### Maximum Tolerated Dose

To determine maximum tolerated dose (MTD), Alphataxin was delivered *via* oral gavage to 8-wk-old female CD1-1GS mice by AJES Life Sciences, (Stony Brook Univ., Stony Brook, NY; IACUC# 764665-4). The dosing study included the following 5 arms with 4 mice/arm: vehicle control (DPBS), Alphataxin (100 mg/kg), Alphataxin (200 mg/kg), Alphataxin (300 mg/kg), and Alphataxin (400 mg/kg). Beginning with the lowest dose, Alphataxin was delivered by oral gavage to a single mouse, and after 2 hrs observation, if the treated mouse appeared to suffer no signs of distress, Alphataxin at the same dose was administered to the other 3 mice in that dosing arm. After 24 hrs observation with no signs of distress, the next dose increment was administered to a separate set of 4 mice and monitored in the same manner. Mice were weighed daily and observed for 12 days after dosing. The highest dose tested indicating no signs of distress or loss of weight was determined to be the MTD.

### Flow Cytometry

Surface staining on whole blood in clinical trial NCT01731691 was previously described (17).

Two protocols were used to enumerate circulating T cells in mice: whole-blood staining at Stony Brook Univ., and lysed-blood staining at Charles River. For whole-blood staining, antibodies were added to whole blood to detect CD3e^+^, CD4^+^, and CD8a^+^ using a mouse T lymphocyte subset antibody cocktail with isotype controls (BD Biosciences, Cat. # 558431) as recommended by the manufacturer. Staining for flow cytometry and statistical analysis was performed by αlpha-1 Biologics, and the samples were independently assessed by the Flow Cytometry Laboratory, Stony Brook Hospital, using a BD LSR Fortessa instrument. Cells in the CD3^+^ T cell gate were evaluated to detect CD4^+^, CD8^+^, and DP T cells using BD FACSDiva software.

For lysed-blood staining, mouse blood samples were processed by adding a 10X volume of room temperature ammonium-chloride-potassium (ACK) lysis buffer to blood. The RBC lysis reaction was quenched by the addition of a 10X volume of cold PBS, and samples were washed and re-suspended at 2 x 10^7^ cells/ml 100 μl of single cell suspensions. Cells were stained for 30 minutes at 4°C with 100 μl of the reconstituted Live/Dead Aqua (Life Technologies) following manufacturer’s instructions. Fc receptors were blocked using TruStain Fc (Biolegend) in 50 μl volume for 5-10 min on ice prior to immunostaining. Cells were stained for 30 min at 4°C with 100 μl of Staining Buffer containing anti-CD3 (BioLegend, Cat. # 100228), anti-CD4 (BD Biosciences, Cat. # 563790), and anti-CD8 (BioLegend, Cat. # 100708) antibodies. Isotype-control antibodies were used as negative staining controls when deemed necessary. All data were collected on a Fortessa (BD) and analyzed with FlowJo software (Tree Star). The gating strategy was determined by initial gating on singlets (FSC-H *vs*. FSCA), and then live cells based on Live/Dead Aqua viability staining. Percentages of cell populations were determined according to the parent cell gate.

### Bioluminescence Intensity (BLI) Imaging of Tumor Size and Metastasis

Dorsal images (unshielded) were acquired to obtain whole body bioluminescence on Days 6, 12, 15, 20, 25, 32, 39 and 46. In addition, on Days 6, 15, 20, 32, 39 and 46 images of metastases to the lung were obtained by acquiring ventral images shielded below the chest with a matt lexan sheet cut out to block luminescence signal from the primary implantation site. Luciferase activity was measured in live animals using IVIS^®^ SpectrumCT (PerkinElmer, Inc., MA) equipped with a CCD camera (cooled at -90°C), mounted on a light-tight specimen chamber. On the day of imaging, 0.22 μm-filter-sterilized VivoGlo™ D-Luciferin substrate (Promega Corporations, WI) dissolved in PBS (150 mg/kg) was split into two IP injections in a dosing volume of 10 ml/kg based on body weight. Mice were placed in an anesthesia induction chamber (2.5-3.5% isoflurane in oxygen). Sedated animals were then immediately moved into prone and supine positions in anesthesia nosecones in the imaging chamber equipped with a stage heated at physiological temperature for image acquisition 10 min post-luciferin substrate injections. The light emitted from the bioluminescent cells was detected, digitalized, and electronically displayed as a pseudo-color overlay onto a gray scale photographic image acquired immediately prior to bioluminescent imaging to allow for anatomical localization of the signal. Data were analyzed using Living Image software 4.5.1. (PerkinElmer, Inc., MA). Flux equaling the radiance (photons/s) in each pixel summed or integrated over the region of interest area (cm^2^) x 4π was used to report quantifiable bioluminescent signal reflecting tumor burden. Regions of interest were drawn around each mouse image, and flux was quantified and reported as 10^6^ photons/s. For quantification purposes, animals with minimal signal were imaged again in the absence of bright animals to avoid interference in the signal acquisition.

### Statistical Analysis

Means were compared using one-way analysis of variance or Student’s t-test. Medians were compared using one-way Kruskal-Wallis analysis of variance on ranks or the Mann-Whitney rank-sum test. Statistical comparisons and graphs were performed using SigmaPlot software with a significance level of alpha = 0.05 and power of test = 0.8 for all reported comparisons.

The definition of “statistical significance” strictly adopted in the scientific literature is that the probability of two treatment effects being shown to be different if they are not different is less than 5% (P< 0.05), given the null hypothesis that there is no difference, *i.e.*, when two treatment effects are not different, the probability of their being wrongly shown to be different is less than 5% (P< 0.05) (31). This definition is dependent on sample size, and the smaller the sample size, the less likelihood that a statistically significant result may be obtained despite genuine treatment differences having practical statistical importance (32–34). In the statistical literature, some *P* values (0.05>*P*<0.1) are interpreted as having practical statistical importance that the two treatments are genuinely different although these are not interpreted as statistically significant using the definition adopted in the scientific literature (34).

## Results

### α1PI Treatment Changes Subpopulations of T Cells in Humans

**Figure 1:**
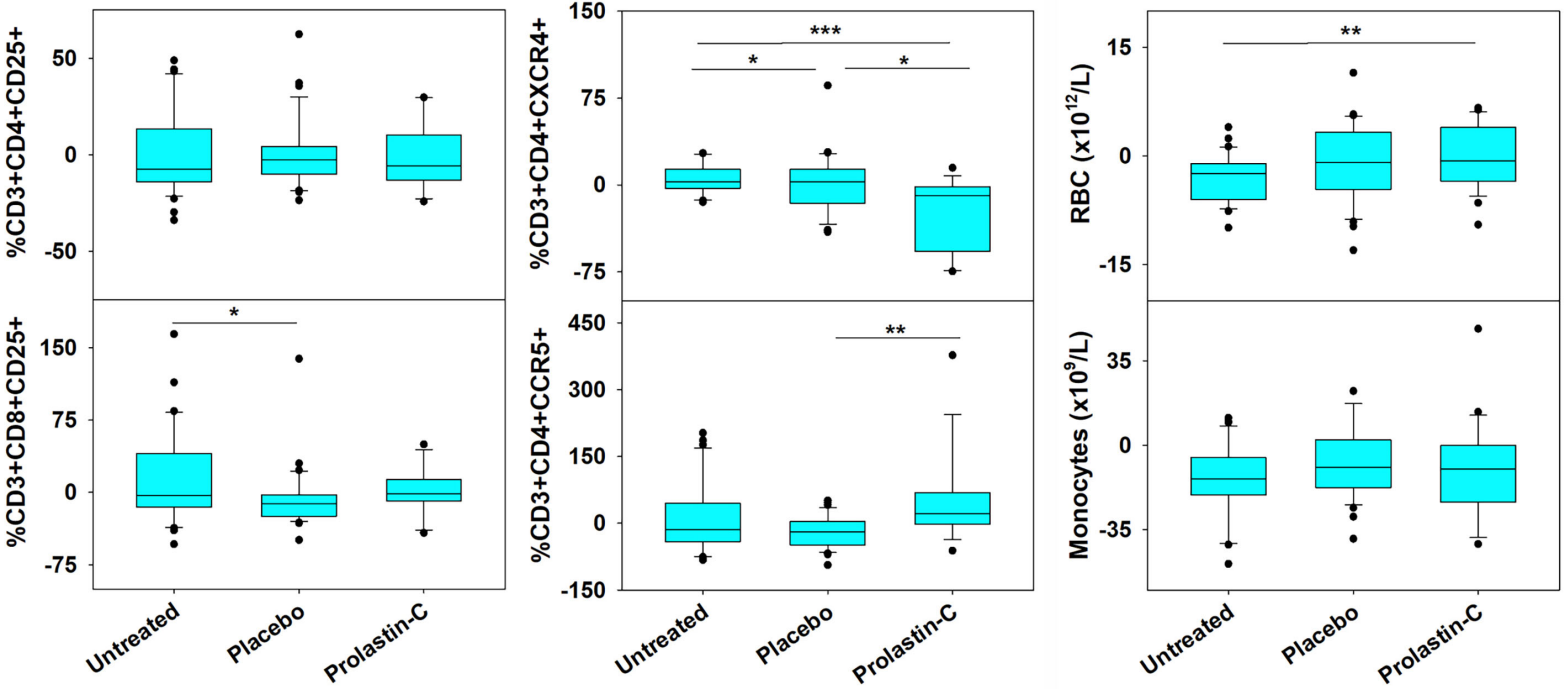
Changes in RBC and the Number of Circulating T Cell Subpopulations in HIV-1 Infected Subjects Treated with Exogenous α1PI. The number of longitudinal observations from the 4 uninfected, untreated subjects, 5 placebo-treated, and 3 Prolastin-C-treated subjects are n = 32, n = 36, and n = 14, respectively. Blood was collected weekly and analyzed by flow cytometry for CD3, CD4, CD8, CD25, CD45RA, CD45RO, CXCR4, CCR5, VLDL receptor, CD91, and CD34. Data (% of lymphocyte gate) represent % change from baseline calculated as (Baseline -Treatment)/Baseline). Red blood cells (RBC) and monocytes represent absolute values as determined from complete blood count. Askerisks designate significant differences (*P < 0.05, **P < 0.01, ***P < 0.001).

As compared with placebo, we previously showed that α1PI treatment significantly elevated CD4/CD8 ratios (17). To determine whether changes to specific subpopulations of CD3^+^ T cells occur following treatment with the α1PI plasma protein, extended phenotypic analysis was examined by flow cytometry on clinical trial blood samples. It was found that α1PI treatment increased the proportion of CD3^+^CD8^+^ CD25^+^ T cells in HIV-1 infected subjects (*n*=14) to a degree that they were not different from uninfected, untreated subjects (*n*=32) whereas placebo-treated HIV-1 infected subjects (*n*=36) exhibited a significantly lower proportion from uninfected, untreated subjects (**Figure 1**). In contrast, α1PI treatment did not significantly change the proportion or number of CD3^+^CD4^+^ CD25^+^ T cells. Interestingly, as compared with uninfected, untreated subjects, α1PI treatment significantly decreased the proportion of CD3^+^CD4^+^CXCR4^+^ T cells (P<0.001, *n*=32 and *n*=14, respectively) and significantly increased the proportion of CD3^+^CD4^+^CCR5^+^ T cells (*P*<.01, *n*=32 and *n*=14, respectively) (**Figure 1**). While α1PI treatment has not been found to change the numbers of myeloid lineage cells, the number of red blood cells (RBC) were increased as compared with untreated, uninfected individuals (*P*=0.008, n=23 and n=32, respectively) (**Figure 1**). Thus, these data and previously published data suggest that α1PI treatment affects RBC numbers as well as some subpopulations of T cells more so than others (17, 24).

### Alphataxin Increases Circulating T Cell Numbers

**Figure 2:**
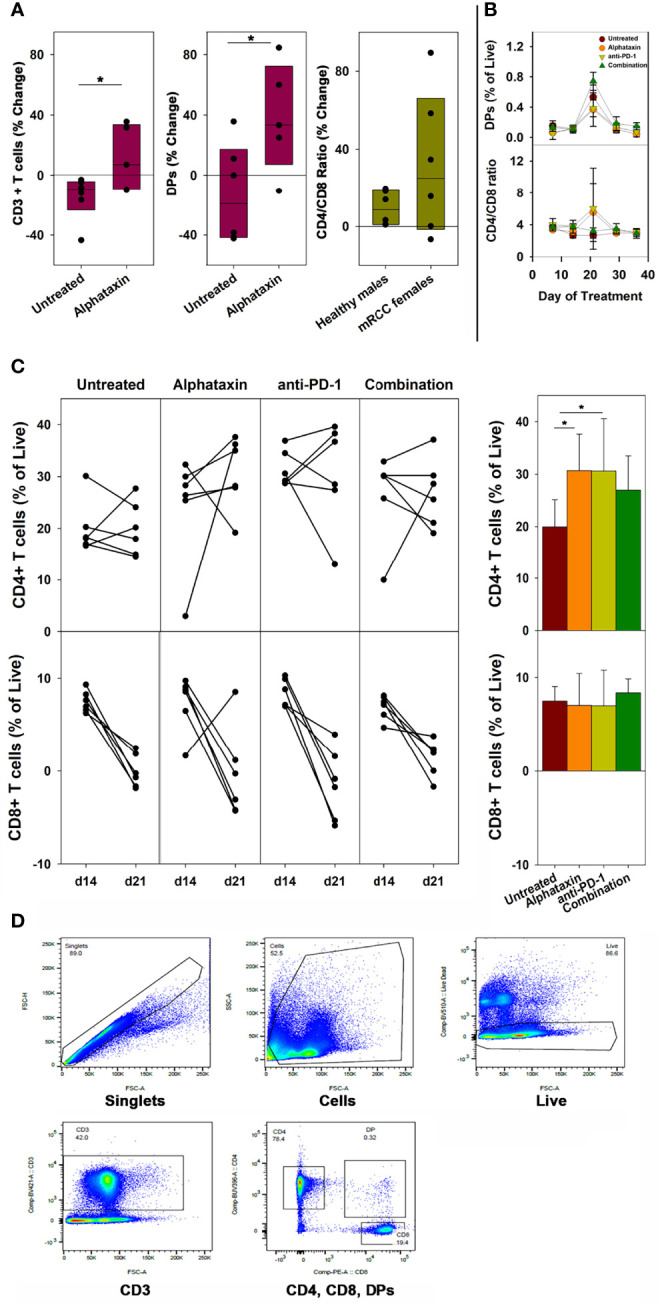
Changes in the Number of Circulating CD3^+^ T Cells, CD4^+^ T Cells, CD8^+^ T Cells, DP Cells and Ratio of Circulating CD4/CD8 Cells in Mice Treated with Alphataxin. **(A)** Blood samples from healthy C57BL/6 mice treated for 2 weeks with Alphataxin (5 mg/kg) or vehicle control (DPBS) were analyzed by flow cytometry to detect circulating CD3^+^ T cells % change from baseline (*P* < 0.05, n = 6) and CD4^+^CD8^+^ DP cells % change from baseline (*P* < 0.04, n = 6). In Alphataxin treated mice, CD4/CD8 ratios (% change from baseline) from healthy C57BL/6 male mice did not differ from those from tumor-bearing female BALB/c mice (*P* = 0.589, n = 6 each). The mean % change from baseline was calculated as 100 x [(treatment mean-baseline)/baseline]. **(B, C)** In BALB/c mice orthotopically implanted with 5x10^3^ Renca-GL cells, the changes in CD4^+^ and CD8^+^ T cell numbers between day 14 and day 21 of treatment for each animal is depicted. Circulating lymphocytes from the untreated (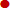), Alphataxin-treated (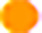), anti-PD-1 antibody-treated (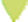), and combination-treated (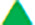) groups were enumerated by flow cytometry gated on live CD3^+^ T cells. After 21 days of treatment, the number of circulating CD4^+^CD8^+^ DP cells was significantly greater in the combination treatment group than in the other treatment groups (*P* = 0.002, n = 6/arm). After 21 days of treatment, the ratio of circulating CD4/CD8 cells was significantly increased in Alphataxin- and anti-PD-1-treated mice compared with the combination-treated and untreated mice (*P* < 0.001, n = 6/arm). After 21 days of treatment, the number of CD4^+^ T cells were significantly increased in Alphataxin- and anti-PD-1-treated mice as compared with untreated mice (*P* = 0.01 and *P* = 0.04, respectively, n = 6/arm). There was no change in the number of CD8^+^ T cells in any group (*P* = 0.81). **(D)** An example of gating strategy from analysis of an animal in the combination treatment arm. The numbers of and % of lymphocytes were normally distributed, and differences among groups were tested by ANOVA and t-test. The CD4/CD8 ratios were not normally distributed, and differences were tested by Kruskal-Wallis one-way ANOVA on ranks. Means and standard deviations are shown. Asterisks designate statistically significant differences (*P < 0.05).

To examine whether the small molecule drug Alphataxin exhibited effects similar to the α1PI plasma protein on circulating T cell numbers, we examined CD4^+^ T cells and CD8^+^ T cells by flow cytometry at baseline and during Alphataxin treatment in non-tumor-bearing male C57BL/6 mice. It was not possible to obtain sufficient blood from mice to parallel the analysis of subpopulations in humans; however, the numbers of circulating CD3^+^ T cells and DPs significantly increased (*P*<0.05, *n*=6/arm and *P*<0.04, *n*=6/arm, respectively) after 2 weeks of daily oral delivery of Alphataxin (5 mg/kg) as compared with the vehicle control (**Figure 2A**). There was no significant difference in CD4/CD8 ratios between healthy non-bearing male C57BL/6 mice and the tumor-bearing female BALB/c mice (described below) (*P*=0.589, *n*=6 each); however, it is not possible to determine from these studies whether the absence of difference between male and female mice is an effect of sex, strain, or tumor bearing status of the mice (**Figure 2A**).

In female BALB/c mice implanted orthotopically with 5 x 10^3^ Renca-GL cells, after 21 days and after 29 days of treatment, the number of circulating DP singlets was significantly greater in the combination treatment arm than in the monotherapy arms (*P*=0.002, *n*=6/arm and *P*=0.02, respectively) (**Figure 2B**). This timing is consistent with thymopoiesis which requires 21 days in mice (35). In contrast, in Alphataxin and anti-PD-1 monotherapy arms, after 21 days of treatment, the circulating CD4/CD8 ratio was significantly higher than in the combination-treated and untreated groups (*P*<0.001, *n*=6/arm), but this difference was not significant after 29 days of treatment (*P*=0.13) (**Figure 2B**). Since the greatest increase in CD4/CD8 ratio was found to occur between 14 and 21 days of treatment (**Figure 2B**), the change in CD4^+^ and CD8^+^ T cells was examined for each animal between days 14 and 21 (*n*=6/arm) (**Figure 2C**). After 21 days of treatment, the numbers of circulating CD4^+^ T cells in mice treated with Alphataxin or anti-PD-1 monotherapy were significantly increased compared with untreated mice (*P*=0.01 and *P*=0.04, respectively, *n*=6/arm) (**Figure 2C**). In mice treated with combination therapy, CD4^+^ T cells were increased as compared with untreated mice in a manner having practical statistical significance (*P*=0.066, *n*=6/arm) (**Figure 2C**). There was no difference in the number of circulating CD8^+^ T cells among the treatment arms (*P*=0.81) (**Figure 2C**). An example of gating strategy is depicted (**Figure 2D**). These data confirm the influence of Alphataxin as a surrogate for α1PI to elevate the number of circulating CD4^+^ T cells in relation to CD8^+^ T cells and to increase the number of immature DPs during thymopoiesis.

### Maximum Tolerated Dose Supports Low Toxicity of Alphataxin

**Figure 3:**
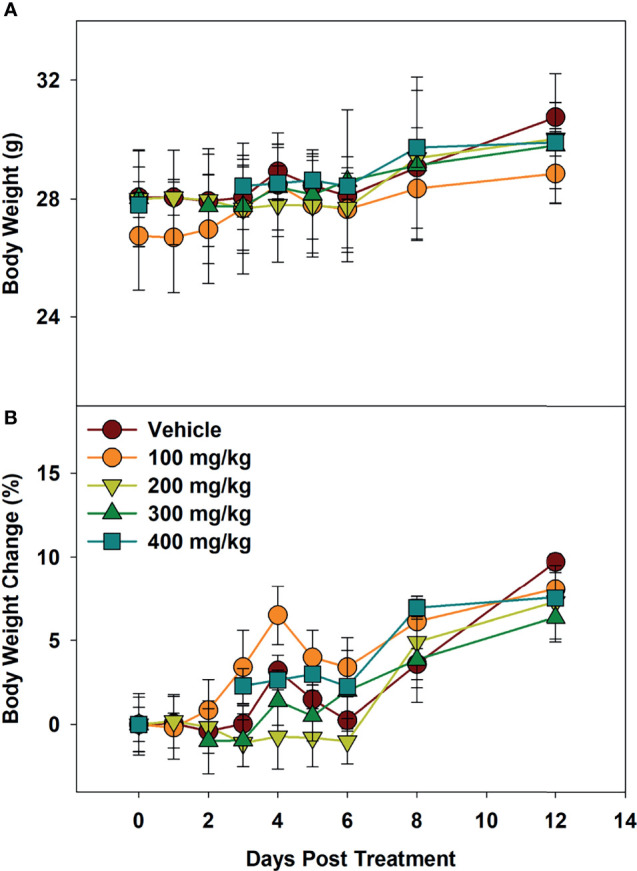
Minimum Tolerated Dose Body Weight Changes. Mice were dosed with increasing concentrations of Alphataxin. **(A)** Body weights after dosing. **(B)** Body weight % change after dosing. The dosing study included the following 5 arms with 4 mice/arm: vehicle control (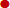), 100 mg/kg (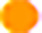), 200 mg/kg (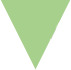), 300 mg/kg (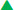), and 400 mg/kg (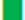). Mice were weighed daily and observed for 12 days after dosing. There was no decrease in body weight with any dose (*P* = 0.39, *n* = 4/arm). The highest dose tested indicating no signs of distress or loss of weight, 400 mg/kg, was determined to be the minimum tolerated dose (MTD).

To determine maximum tolerated dose (MTD), mice were administered Alphataxin and observed for signs of distress or weight loss for 12 days. In the highest dose tested (400 mg/kg), there were no signs of distress or differences in body weight (*P*=0.39, *n*=4/group) (**Figure 3**). These data support evidence that Alphataxin has low toxicity even at doses 40-fold higher than the effective dose (10 mg/kg).

### Alphataxin Suppresses the Growth of Subcutaneously Implanted Renca Cells

**Figure 4:**
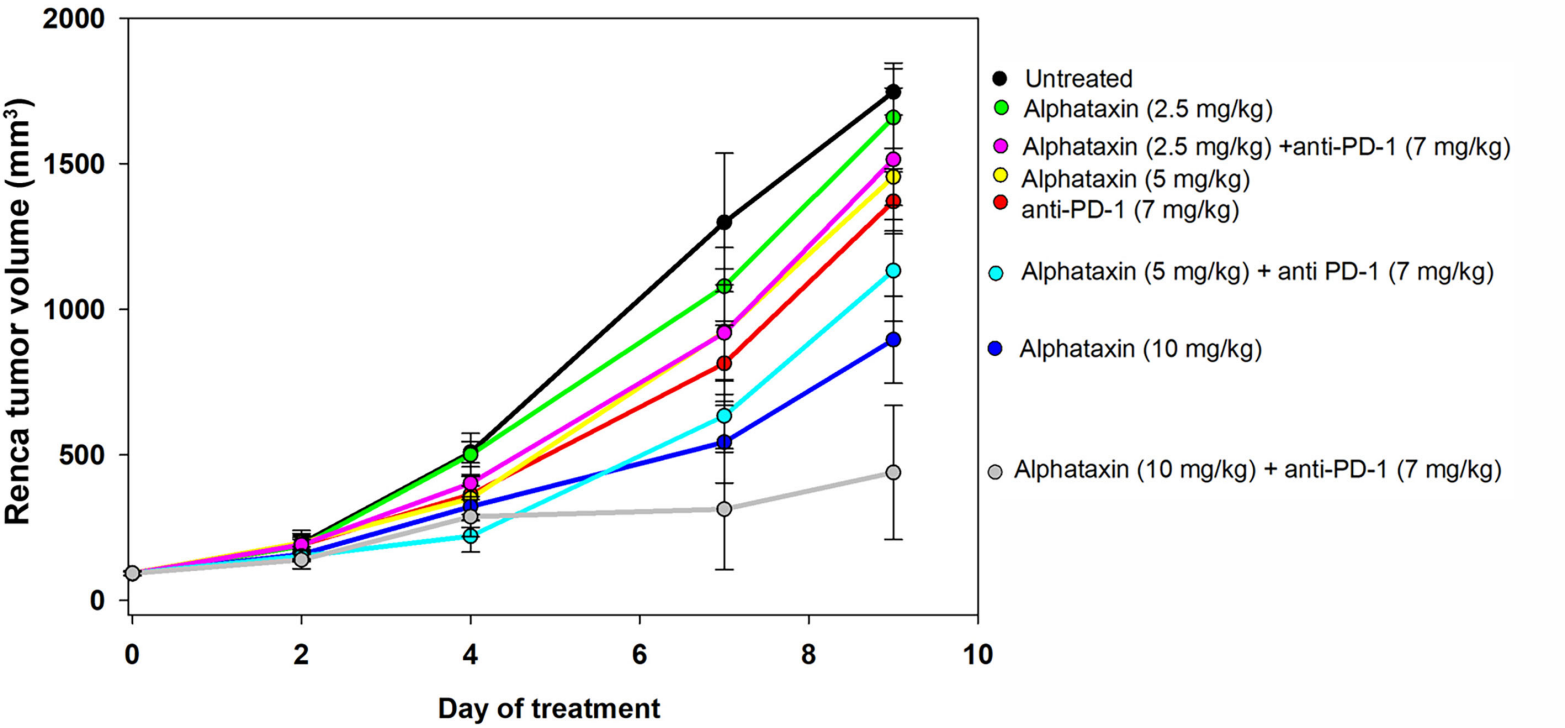
Tumor Growth Curves of Subcutaneously Implanted Tumor Cells. Renca cells (2 x 10^5^) were subcutaneously implanted into 8 female BALB/c mice per group and allowed to grow for 2 weeks before the mice remained untreated (●, n = 7) or were treated with an anti-PD-1 antibody (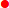, 7 mg/kg, n = 6) *via* IP injection twice per week or with Alphataxin *via* daily oral gavage at a dose of 2.5 mg/kg (●), 5 mg/kg (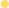), or 10 mg/kg (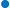) (n = 8/arm). In combination treatment, mice were treated with anti-PD-1 (7mg/kg) *via* IP injection twice per week and with Alphataxin *via* daily oral gavage at a dose of 2.5 mg/kg (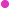), 5 mg/kg (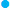), or 10 mg/kg (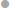) (n = 8/arm). Tumor volume was calculated periodically using calipers. Alphataxin (10 mg/kg) significantly suppressed tumor growth as compared with Alphataxin (5 mg/kg) or Alphataxin (2.5 mg/kg) (P < 0.001, n = 8/arm). Data were normalized to baseline tumor volume and are presented as the means and standard deviations.

Because Alphataxin was found to elevate circulating T cell numbers, we examined whether Alphataxin is an efficacious treatment in murine renal adenocarcinoma, a representative T cell-responsive tumor type. BALB/c mice were subcutaneously implanted with 2x10^5^ Renca cells. Following 2 weeks of tumor growth, the mice were treated with anti-PD-1 (7 mg/kg) and/or varying doses of Alphataxin for a total of 3 weeks. At 10mg/kg, Alphataxin was found to more effectively decrease tumor growth than 5 mg/kg, and the 2.5 mg/kg was not effective (*P*<0.001, *n*=8 mice/arm) (**Figure 4**). Alphataxin (5 mg/kg or 10 mg/kg) as monotherapy were as effective or more effective, respectively, than anti-PD-1 (7 mg/kg) as monotherapy, and this effect was enhanced when Alphataxin (5 mg/kg or 10 mg/kg) was combined with anti-PD-1 (7 mg/kg) immunotherapy. Thus, 5 mg/kg or 10 mg/kg were considered effective doses of Alphataxin for inhibiting growth of subcutaneous renal adenocarcinoma tumors in mice, and these doses were used in orthotopic tumor models. While a three 2-fold dose curve is limited in dose numbers, by comparing percent inhibition of tumor growth, it appeared that combining Alphataxin with anti-PD-1 antibody had an additive effect, as opposed to synergistic effect, which was expected because the two drugs target separate biological pathways; more sophisticated methods would be informative using a larger set of dose curves (**Table 1**) (36).

**Table 1:** Additive *vs* Synergistic Effects on Tumor Growth by Anti-PD-1 and Alphataxin.

Treatment	Mean Inhibition^a^	Coefficient of Variation^b^	Sum of effects^c^	Ratio of effects^d^
Untreated	0%			
anti-PD-1 (7mg/kg)	21.5%	2.12%		
Alphataxin (2.5mg/kg)	5.0%	13.19%		
Alphataxin (5mg/kg)	16.7%	2.07%		
Alphataxin (10mg/kg)	48.7%	1.08%		
anti-PD-1 + Alphataxin (2.5mg/kg)	13.3%	6.99%	26.5%	0.501
anti-PD-1 + Alphataxin (5mg/kg)	35.1%	1.9%	38.2%	0.919
anti-PD-1 + Alphataxin (10mg/kg)	74.8%	1.09%	70.2%	1.066

a % Inhibition of tumor growth for each animal was calculated from data presented in **Figure 2** on day 9 of treatment as (untreated – treated)/untreated*100. Mean % inhibition normalized to untreated animals is depicted for each group (n = 6 mice/group).

b Coefficient of variation (Std. Dev/Mean) is provided as a measure of variation of normalized data.

c The additive effect was calculated as the sum of % inhibition by anti-PD-1 (7 mg/kg) and % inhibition by Alphataxin at each dose. The sum of individual drug effects was comparable to the combination treatment effects.

d The synergistic effect was calculated as the ratio of mean % inhibition in the combination treatment groups divided by the sum of individual drug effects. In the absence of synergy, the ratio would be expected to be 1, and if synergy exists, the ratio would be expected to be greater than 1. The ratio was not appreciably greater than 1.

### Alphataxin Combined With Anti-PD-1 Immunotherapy Suppresses the Orthotopic Tumor Growth of Renca Cells

**Figure 5:**
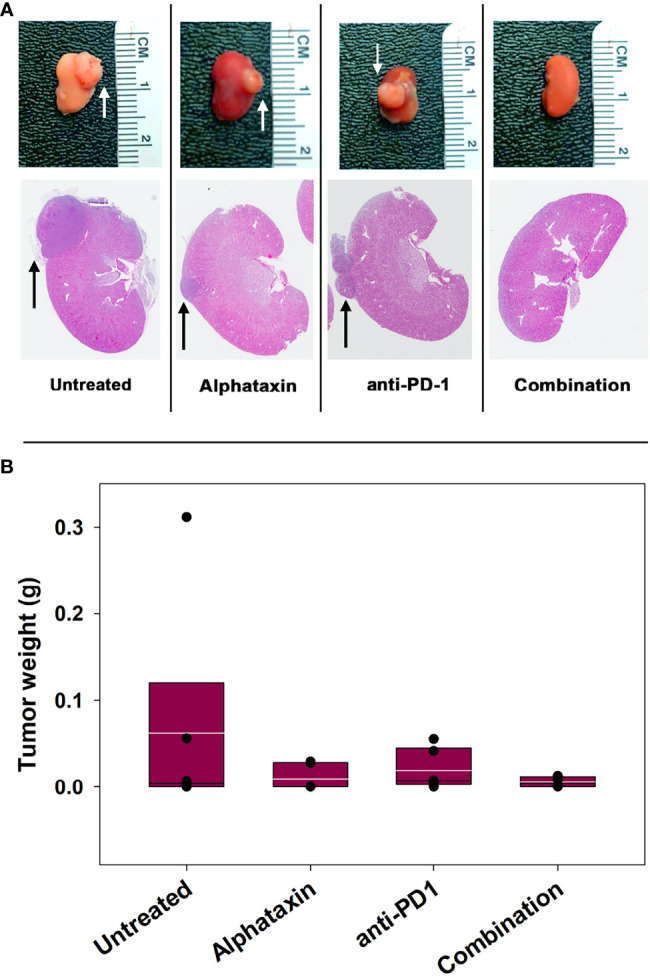
Tumor Growth was Inhibitied by Combination Treatment with Alphataxin and an Anti-PD-1 Antibody. Mice were orthotopically implanted with 5x 10^2^ renal carcinoma cells, and treatment was initiated 7 days later. **(A)** Excised kidneys were compared for gross morphology (row 1) and by H&E staining (row 2). Images were captured at identical magnification, and representative images at 1X are depicted with tumors indicated by arrows. **(B)** The tumor weight of each animal was calculated as the difference between the weight of the left tumor-bearing kidney and that of the right non-tumor-bearing kidney from each mouse assuming that the left and right kidneys are symmetrical and have approximate equal weights other than the added tumor weight. Tumor weights were not normally distributed. Medians, depicted by black lines within box plots, were compared using Kruskal-Wallis ANOVA of ranks (n = 6/arm). White lines within box plots depict means.

The subcutaneous tumor model provides a less costly and shorter time frame for frequent measurement to assess optimal dosing than the orthotopic tumor model. However, the subcutaneous model is not relevant to metastasis and provides less circulatory access to drugs than the orthotopic model. We next examined how combination treatment with Alphataxin and anti-PD-1 immunotherapy affects the orthoptic tumor growth using various numbers of implanted Renca cells and dosages of Alphataxin. Female BALB/c mice were orthotopically implanted with 5 x 10^2^ Renca cells, and tumor cells were allowed uninhibited growth for 7 days after which mice were treated for 21 days with anti-PD-1 (7 mg/kg) and/or Alphataxin (5 mg/kg). Kidneys were removed from euthanized mice, and tumors were visibly detected in untreated, Alphataxin-treated, and anti-PD-1-treated mice (**Figure 5A**). In contrast, there were no detectable tumors in the 6 mice that received combination therapy with Alphataxin and anti-PD-1. Due to the large variation in tumor size in untreated mice, median tumor weight in untreated and treated mice were not significantly different (**Figure 5B**). However, the lack of detectable tumors in the combination treatment arm compared to the untreated arm clearly demonstrated that combination treatment produced tumor regression or dramatically decreased tumor growth.

**Figure 6:**
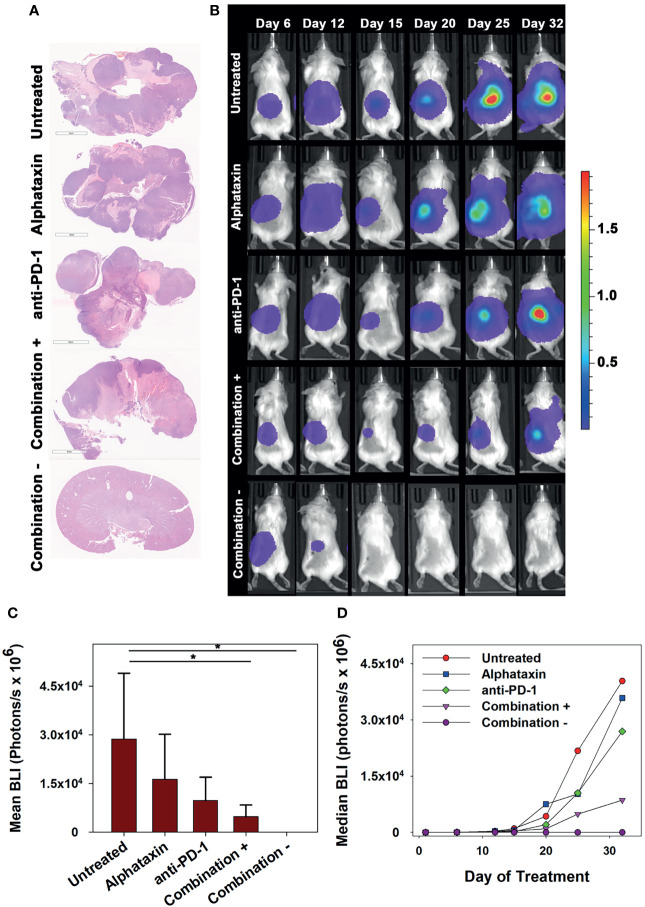
Long-term Remission Achieved by Treatment with Alphataxin and an Anti-PD-1 Antibody. Mice were orthotopically implanted with 5x 10^3^ Renca-GL cells, and treatment was initiated the following day. **(A)** Kidneys were excised at the time of euthanization and stained with H&E. Images were captured at identical magnification, and representative images at 1X are shown. **(B)** Longitudinal dorsal images of the orthotopic kidney tumor growth of mice. A representative animal that showed similar bioluminescence with at least 2 other animals within the same treatment arm after 25 days of treatment was selected from each treatment arm. The mice in the combination treatment arm were bifurcated into 2 groups, those with tumors (“Combination +”, 4^th^ row, n = 4) and those without tumors (“Combination −”, 5^th^ row, n = 2). The luminescence color scale represents photons/sec/cm^2^/steradian x 10^9^ with minimum 4.48e6 and maximum 19.4e9. **(C)** As compared with untreated mice and monotherapy, after 25 days of treatment the tumor volumes (BLI) were significantly smaller in mice receiving combination treatment with tumors (*P* = 0.05, n = 4) and without tumors (*P* = 0.02, n = 2). After 25 days of treatment, there were no significant differences between untreated and Alphataxin-treated mice (*P* = 0.24, n = 6/arm) or between untreated and anti-PD-1-treated mice (*P* = 0.06, n = 6) although the difference between untreated and anti-PD-1-treated mice have practical statistical importance. Means and standard deviations are depicted. **(D)** Median longitudinal tumor growth demonstrates that as compared with untreated animals (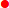), Alphataxin (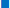) and anti-PD-1(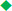) individually impeded tumor growth and in combination + animals (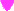), substantially impeded tumor growth while in combination – animals (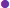), tumor growth regressed. Asterisks designate statistically significant differences (*P < 0.05).

To allow frequent monitoring of tumor growth and to distinguish tumor regression from decreased tumor growth, Renca cells were used which stably express green fluorescent protein (GFP) and firefly luciferase (Renca-GL) (29). Female BALB/c mice were orthotopically implanted with 5 x 10^3^ Renca-GL cells. Due to the large number of implanted tumor cells, treatment was initiated after 1 day of tumor growth with anti-PD-1 antibody (7 mg/kg) and/or Alphataxin (10 mg/kg). After 25 days of treatment, tumor size as indicated by BLI was significantly lower in the mice in the combination treatment arm than in those in untreated or monotherapy treatment arms (*P*=0.023, n=6/arm) (**Figures 6A, B** **)**.

After 32 days of treatment, untreated mice had become moribund and were euthanized. At that time, there were detectable tumors in all untreated, anti-PD-1-treated, and Alphataxin-treated mice as indicated by BLI. In contrast, there were no tumors in 2 of the mice in the combination treatment arm (**Figure 6**). After 32 days of treatment, the combination treatment arm was bifurcated into tumor-bearing (n=4) or tumor-free (n=2) groups for comparisons; however, it should be noted that the 2 surviving mice in the combination treatment arm with 100% tumor regression had shown positive evidence of Renca-GL cells localized in the kidney on days 6 and 12 following implantation (**Figures 6B, C**). After 32 days of treatment, in the tumor-bearing mice in the combination treatment group, tumor volume was 81% smaller than that in untreated mice (*P*=0.1, *n*=4 combination-treated *vs n*=6 untreated mice). Longitudinal comparison of median growth between groups shows that as compared with untreated mice, monotherapy impeded tumor growth and combination treatment substantially impeded or regressed tumor growth (**Figure 6D**).

At the time of euthanization, blood was collected for complete blood cell count and differential. Analysis showed no significant changes in any blood cell counts except for lymphocytes and RBCs (**Table 2**). Lymphocytes were significantly increased in the combination-treated mice (*P* < 0.001), Alphataxin-treated mice (*P* < 0.001), and anti-PD-1-treated mice (*P*=0.002) as compared with untreated mice. RBCs, as in α1PI treated humans, were significantly increased in combination-treated mice (*P*=0.005).

**Table 2:** Complete Blood Count and Differential.

	Untreated (n = 5)	Alphataxin (n = 3)	anti-PD-1 (n = 3)	Combination (n = 2)

**WBC (K/µL)**	10.12 ± 7.44	8.46 ± 4.5	17.06 ± 15.67	11.67 ± 2.86
**NEU (K/µL)**	7.83 ± 7.05	6.51 ± 3.4	13.39 ± 13.01	3.11 ± 0.82
**LYM (K/µL)**	1.88 ± 0.62	1.36 ± 0.96	2.2 ± 1.36	8.23 ± 1.80***
**MONO (K/µL)**	0.116 ± 0.12	0.16 ± 0.11	0.28 ± 0.15	0.08 ± 0.08
**EOS (K/µL)**	0.078 ± 0.05	0.1 ± 0.05	0.18 ± 0.2	0.22 ± 0.22
**BASO (K/µL)**	0.032 ± 0.03	0.10 ± 0.10	0.24 ± 0.18	0.05 ± .05
**RBC (M/µL)**	5.272 ± 1.22	6.61 ± 0.95	4.74 ± 1.94	10.49 ± 0.07**
**HGB (g/dL)**	8.14 ± 1.81	10.1 ± 1.66	7.7 ± 3.2	15.3 ± 0.1
**HCT (%)**	29.02 ± 5.71	36.17 ± 4.69	27.77 ± 11.49	48.9 ± 0.3
**MCV (fL)**	55.46 ± 2.43	54.83 ± 1.17	59 ± 4.47	46.6 ± 0.5
**MCH (pg)**	15.5 ± 0.21	15.3 ± 0.35	16.17 ± 0.35	14.6 ± 0.2
**MCHC (g/dL)**	28 ± 0.89	27.9 ± 1.18	27.47 ± 1.72	31.4 ± 0
**PLT (K/µL)**	498.8 ± 220.58	534 ± 374.5	499.67 ± 156.56	976.5 ± 5.5
**LEUKOCYTES (%)**	1.66 ± 1.96	2.67 ± 0.45	3.8 ± 1.73	0 ± 0

At moribundity or on the last day of the study, full blood volume (0.175 ml per mouse) was collected and shipped to Antech Diagnostics to conduct a complete blood cell count with manual differential. Groups were compared by ANOVA, and those with significant differences are denoted.

^***^Lymphocytes were significantly increased in the combination-treated mice as compared with untreated (P < 0.001), Alphataxin-treated (P < 0.001), and anti-PD-1-treated mice (P = 0.002).

^**^RBCs were significantly increased in the combination-treated mice as compared with untreated (P = 0.005), Alphataxin-treated (P = 0.038), and anti-PD-1-treated mice (P = 0.005).

Animals showed no adverse effects in any treatment arm. Importantly, no mice in either the Alphataxin or combination treatment arms had to be euthanized due to tumor ulceration, whereas some animals in the other groups had to be euthanized for this reason (untreated, *n*=1; anti-PD-1, *n*=2).

### Combination Treatment With Alphataxin and Anti-PD-1 Immunotherapy Suppresses Lung Metastasis

**Figure 7:**
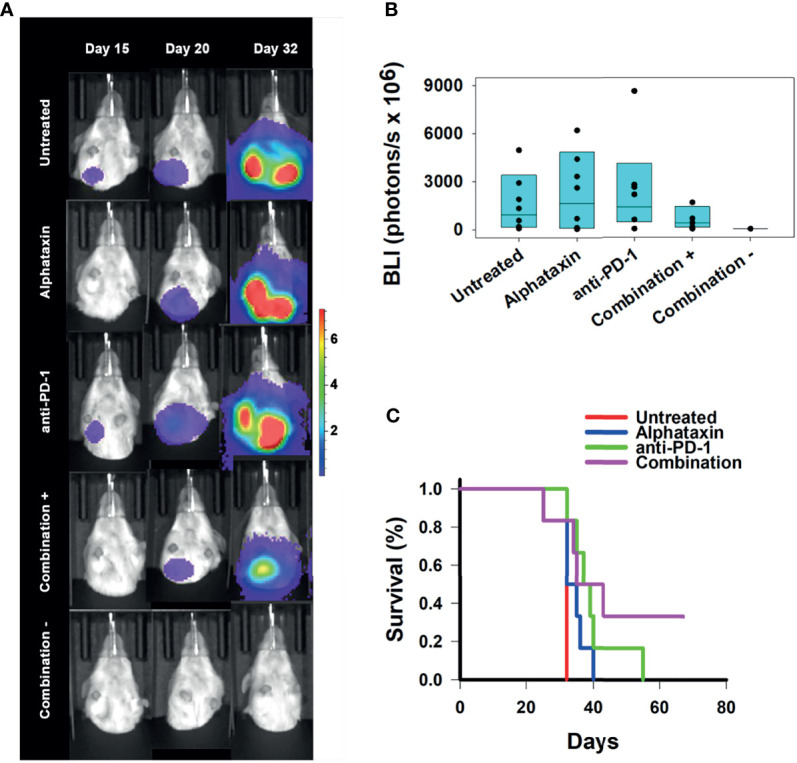
Suppression of Lung Metastasis Achieved by Treatment with Alphataxin and an Anti-PD-1 Antibody. Mice were orthotopically implanted with 5x 10^3^ Renca-GL cells, and treatment was initiated the following day. **(A)** Longitudinal shielded (ventral) images of the metastatic lung tumor growth. A representative animal that showed similar bioluminescence with at least 2 other animals within the same treatment arm after 20 days of treatment was selected from each treatment arm. The mice in the combination treatment arm were bifurcated into 2 groups, those with kidney tumors (“Combination +”, 4^th^ row, n = 4) and those without kidney tumors (“Combination −”, 5^th^ row, n = 2). The luminescence color scale represents photons/sec/cm^2^/steradian x 10^7^ with minimum 4.8e5 and maximum 7.29e7. **(B)** After 32 days of treatment, there was a 4-log difference in the 25% median lung BLI between the combination treatment arm (2.9x10^6^ photons/s) and untreated arm (2.6x10^10^ photons/s) and a 1-log difference in the 75% median value between the combination treatment arm (8.4x10^9^ photons/s) and the untreated arm (5.6x10^10^ photons/s), which clearly demonstrates that the combination treatment dramatically decreased metastasis with practical statistical importance. The metastatic tumor volumes were not normally distributed, and the medians are shown within each box plot with individual lung BLI values. **(C)** Average survival of mice in the combination treatment arm (violet, 45 days) was significantly greater (*P* = 0.02) than that in the untreated (red, 32 days), Alphataxin (blue, 33 days) or anti-PD-1 (green, 40 days) treatment arms. Increased life span *versus* untreated mice (Treatment/Untreated*100% - 100%) was 22 days in combination-treated mice (*P* < 0.01), 19 days in anti-PD-1-treated mice (*P* < 0.05), and 5 days in Alphataxin-treated mice. All untreated mice were euthanized after 32 days due to being moribund. In contrast, two combination-treated, healthy mice exhibiting no evidence of tumors were euthanized after 67 days to end the study.

To determine whether any of the treatment regimens could suppress lung metastasis, lung BLI was measured. Lung metastasis was not substantially detected in any treatment arm until day 32 which was the first time that lungs were imaged after day 20 imaging (**Figure 7A**). On day 32, lung metastasis was not statistically different in untreated and combination treatment arms (*P*=0.17), and this was due to the large variation in the untreated, Alphataxin-treated, and anti-PD-1-treated arms (**Figure 7B**). However, a 4-log difference between the 25% median value in the combination treatment arm (2.9x10^6^ BLI) and untreated arm (2.6x10^10^ BLI) and a near 1-log difference in the 75% median value between the combination treatment arm (8.4x10^9^ BLI) and the untreated arm (5.6x10^10^ BLI) clearly demonstrate that combination treatment dramatically decreased metastasis in a manner with practical importance (**Figure 7B**). Finally, when the study was terminated after 67 days, the 2 surviving mice in the combination treatment arm were alive, tumor-free, and healthy (**Figure 7C**). The combination of anti-PD-1 (7mg/kg) and Alphataxin (10 mg/kg) decreased tumor growth or induced tumor regression of the implanted renal adenocarcinoma cells suggesting these doses and treatment schedule are sufficiently efficacious.

The efficacy of treatment on lifespan as compared with untreated mice was assessed. Combination treatment increased survival to 45 ± 7 days (mean ± standard error of mean), which represented a 22% increase in lifespan (*P*<0.05, *n*=6/arm) beyond the 32 days for untreated mice (**Figure 7C**). These data demonstrate that combination therapy with Alphataxin and anti-PD-1 can increase survival and allow mice to achieve durable remission.

### Alphataxin Increases the Number of CD4^+^ TILs

**Figure 8:**
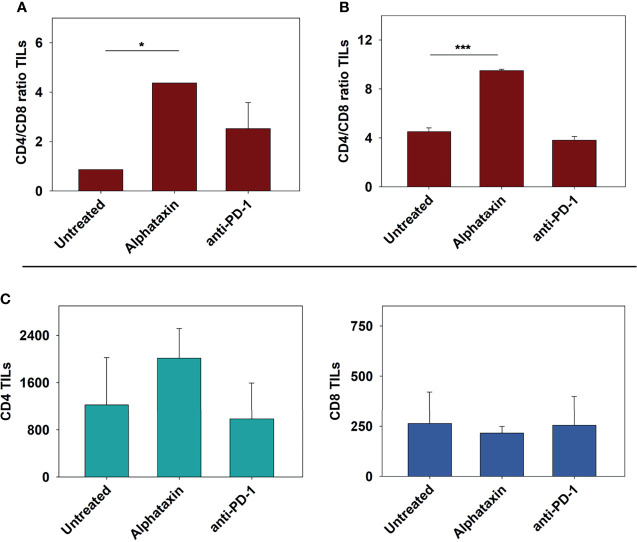
Increases in the Tumor-infiltrating CD4/CD8 Ratio Achieved by Treatment with Alphataxin or an Anti-PD-1 Antibody. Excised kidneys from mice in each treatment arm of (5 x 10^2^ orthotopically implanted Renca cells and 5 x 10^3^ orthotopically implanted Renca-GL cells) were immunohistochemically stained to determine the numbers of CD4^+^ and CD8^+^ TILs which were counted in all the tumor fields of the stained slides. Because the tumors varied in size, yielding large variation in the number of TILs, the CD4/CD8 ratio is depicted to produce more accurate comparisons. **(A)** In the Renca cell orthotopic study, after 21 days of treatment, the tumor-infiltrating CD4/CD8 ratio in Alphataxin-treated animals was significantly greater than that in untreated and anti-PD-1 antibody-treated mice (*P* = 0.04). **(B)** In the Renca-GL study, tumor-infiltrating CD4/CD8 ratio in Alphataxin-treated mice was significantly greater than that in untreated (*P* < 0.001, n = 3) and anti-PD-1-treated mice (*P* < 0.001, n = 3). In anti-PD-1 treatment, CD4/CD8 ratio was significantly greater than in untreated animals (*P* = 0.02, n = 3). Combination treatment is not depicted due to the lack of suitable kidney sectioning. Means and standard deviations are depicted. The CD4/CD8 ratios were not normally distributed and were compared by the Mann-Whitney rank- sum test. **(C)** In the Renca-GL study, there were more CD4^+^ TILs (green bars) in the tumors of Alphataxin-treated mice than in those of anti-PD-1 antibody-treated mice (*P* = 0.086, n = 3), which demonstrates practical statistical importance. The numbers of CD8^+^ TILs (blue bars) were not different among the treatment arms (*P* = 0.94), *n*=3). Note that the scales for the CD4/CD8 ratio and TIL counts are not equivalent in different graphs. TILs were normally distributed and were tested by ANOVA. Asterisks designate statistically significant differences (*P < 0.05, ***P < 0.001).

**Figure 9:**
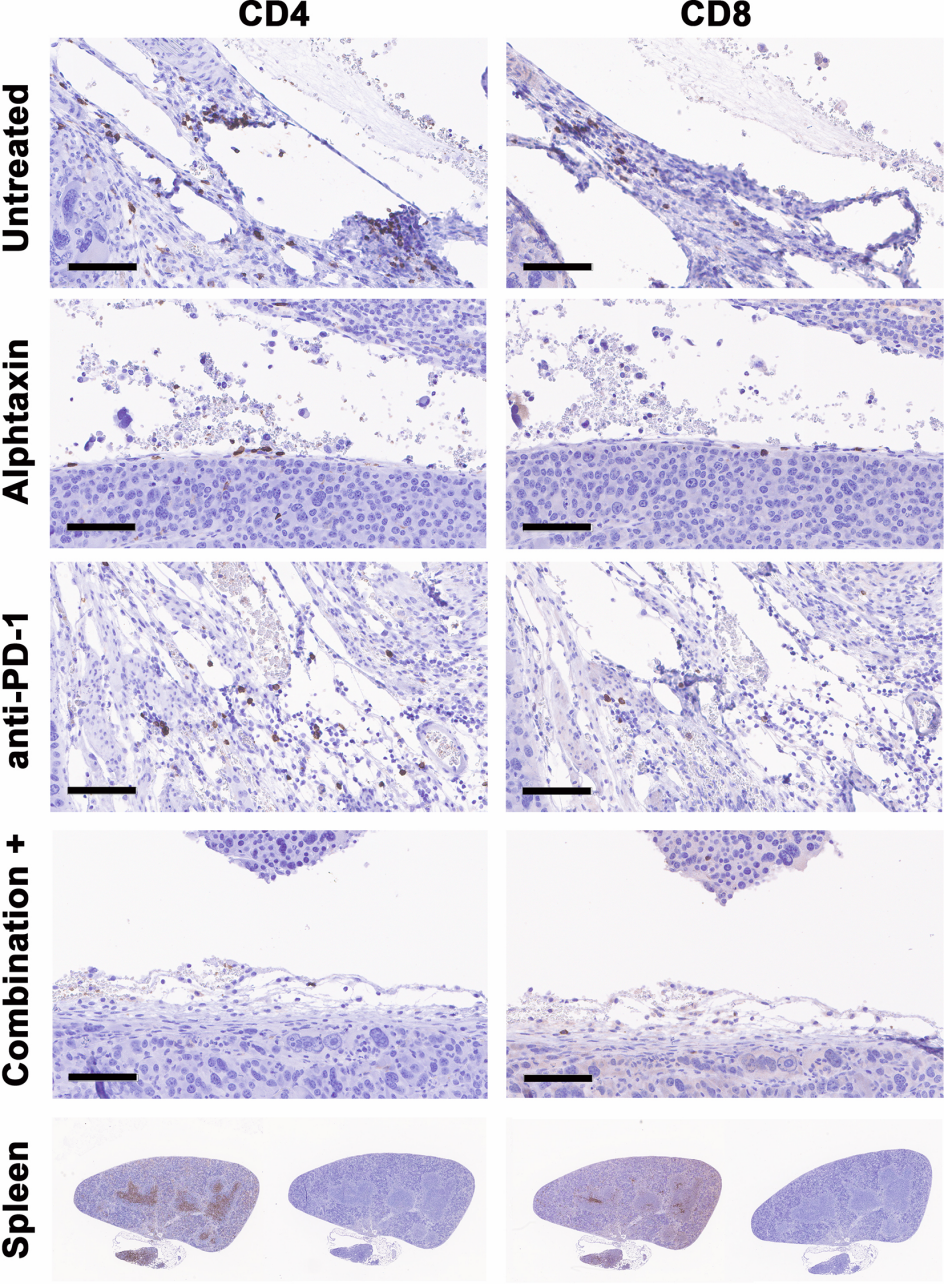
Cryosections from each treatment group stained immunohistochemistry. Representative cryosections depict staining for CD4^+^ T cells (right panels) and CD8+ T cells (left panels) from approximately the same area of tissue. Bars represent 100µm. Positive (right panels) and negative staining controls (left panels) were performed using cryosections from spleen in which CD4+ and CD8^+^ T cells are abundant within germinal centers.

To determine whether the influence of Alphataxin and/or anti-PD-1 treatment might be intratumoral, the number of TILs were counted in kidney tissue cryosections stained for CD4^+^ and CD8^+^ T cells. In mice orthotopically implanted with Renca cells, after 21 days of treatment, the CD4/CD8 ratio in tumors was significantly higher in Alphataxin-treated than in untreated or anti-PD-1-treated mice (*P*=0.04, *n*=2/arm) (**Figure 8A**). In mice orthotopically implanted with Renca-GL cells, CD4/CD8 ratio in tumors was significantly higher in the Alphataxin-treated than untreated or anti-PD-1-treated mice (*P*<0.001, *n*=3/arm) (**Figure 8B**). Additionally, the number of CD4^+^ TILs was higher in Alphataxin-treated than untreated or anti-PD-1-treated mice (*P*=0.086, *n*=3/arm) demonstrating practical statistical importance (**Figure 8C**). In contrast, the number of CD8^+^ TILs did not differ among the treatment arms (*P*=0.94, *n*=3/arm) supporting evidence that both Alphataxin and anti-PD-1 therapy primarily promotes the cytotoxic effectiveness of CD8^+^ TILs as opposed to the number of CD8^+^ TILs as has been reported (**Figure 8C**) (2). Alphataxin treatment increased the CD4/CD8 ratio and the number of CD4^+^ TILs in murine orthotopic kidney tumors. Representative cryosections from each treatment group demonstrate immunohistochemistry staining for intratumoral CD4^+^ and CD8^+^ T cells (**Figure 9**). Positive and negative control staining was performed using spleen tissue in which germinal centers contain abundant lymphocytes.

### Kidney Tumor Growth Is Correlated With Circulating Lymphocyte Levels

To determine whether circulating lymphocytes are related to metastasis and tumor volume, we examined correlations after 21 and 29 days of treatment in all arms (**Table 3**). After 21 days of treatment, inverse correlations were found between kidney tumor size and circulating % CD4^+^ T cells in Alphataxin-treated mice (*r* = −0.80, *P*=0.05, *n*=6/arm); circulating % CD8^+^ T cells in anti-PD-1-treated mice with (*r*= −0.72, *P*=0.105); circulating % CD8^+^ T cells (*r* = −0.83, *P*=0.04) in the combination treatment group (**Table 3**). Importantly, there were no correlations between circulating T cells and metastasis. Thus, decreased tumor size was correlated with increased circulating CD4^+^ T cells in Alphataxin-treated mice and with increased circulating CD8^+^ T cells in anti-PD-1-treated mice.

**Table 3:** Correlation of the Number of Circulating T Cells and Kidney Tumor Size as Indicated by Bioluminescence Intensity (BLI).

T Cells		Untreated			Alphataxin			Anti-PD-1			Combination	
		Day 21	Day 29		Day 21	Day 29		Day 21	Day 29		Day 21	Day 29
		BLI Kidney	BLI Kidney	BLI Lung	BLI Kidney	BLI Kidney	BLI Lung	BLI Kidney	BLI Kidney	BLI Lung	BLI Kidney	BLI Kidney
												
**CD4%**	***r***	**0.51**	**-0.78**	**-0.19**	**-0.80**	**-0.18**	**-0.57**	**-0.19**	**-0.54**	**-0.55**	**-0.57**	**-0.17**
	***P***	**0.31**	**0.07**	**0.72**	**0.05**	**0.77**	**0.31**	**0.73**	**0.27**	**0.26**	**0.24**	**0.79**
	***n***	**6**	**6**	**6**	**6**	**5**	**5**	**6**	**6**	**6**	**6**	**5**
	*** ***											
**CD8%**	***r***	**-0.02**	**-0.76**	**-0.02**	**-0.30**	**-0.18**	**-0.54**	**-0.72**	**-0.66**	**-0.40**	**-0.83**	**-0.19**
	***P***	**0.97**	**0.08**	**0.97**	**0.56**	**0.77**	**0.34**	**0.105**	**0.16**	**0.43**	**0.04**	**0.76**
	***n***	**6**	**6**	**6**	**6**	**5**	**5**	**6**	**6**	**6**	**6**	**5**
	*** ***											
**DP%**	***r***	**0.21**	**-0.65**	**-0.16**	**-0.32**	**0.06**	**-0.58**	**0.02**	**-0.73**	**-0.37**	**-0.57**	**-0.30**
	***P***	**0.69**	**0.16**	**0.76**	**0.53**	**0.93**	**0.30**	**0.96**	**0.10**	**0.47**	**0.24**	**0.63**
	***n***	**6**	**6**	**6**	**6**	**5**	**5**	**6**	**6**	**6**	**6**	**5**
	*** ***											
**CD4/CD8**	***r***	**0.35**	**-0.82**	**0.05**	**0.68**	**-0.28**	**-0.81**	**0.31**	**0.87**	**-0.42**	**0.13**	**0.01**
**Ratio**	***P***	**0.50**	**0.05**	**0.93**	**0.14**	**0.65**	**0.10**	**0.55**	**0.03**	**0.41**	**0.80**	**0.98**
	***n***	**6**	**6**	**6**	**6**	**5**	**5**	**6**	**6**	**6**	**6**	**5**
	*** ***											
**CD3%**	***r***	**-0.24**	**-0.46**	**-0.29**	**-0.07**	**-0.18**	**-0.57**	**-0.24**	**-0.57**	**-0.51**	**-0.63**	**-0.18**
** **	***P***	**0.65**	**0.36**	**0.58**	**0.92**	**0.77**	**0.32**	**0.65**	**0.24**	**0.30**	**0.18**	**0.77**
** **	***n***	**6**	**6**	**6**	**5**	**5**	**5**	**6**	**6**	**6**	**6**	**5**

Red boxes represent negative correlations, green boxes represent positive correlations, and white boxes represent no correlation. The values depicted within each box represent correlation coefficients (r, top row), significance (P, middle row), and number of observations (n, bottom row). Values were normally distributed, and correlations were determined using Pearson Product Moment analysis. While some P values (0.05 < P < 0.1) could not be interpreted as statistically significant, they were interpreted as having statistical practical importance.

## Discussion

Recent evidence demonstrates that CD4^+^ TILs are capable of killing tumor cells and facilitating CD8^+^ TILs (9, 13). Having found that the protein α1PI promotes migration of immature DPs and mature CD4^+^ T cells through tissue in humans, we sought to develop an orally available small molecule, Alphataxin, to act as a surrogate for α1PI in addressing the effects of CD4^+^ T cells on tumor killing function (17). Alphataxin was shown here to effectively elevate CD4^+^ T cell numbers and CD4/CD8 T cell ratios both in circulation and within tumors in non-tumor bearing mice and in two orthotopic renal adenocarcinoma mouse studies. Compared with untreated mice, in one study, combination-treated mice showed complete regression while in another study, combination-treated mice showed either significant tumor growth suppression (81%) or complete tumor regression.

Because Alphataxin was effective as a monotherapy, this suggests that the CD4^+^ TILs were not Tregs, but either CD4^+^ Th1 cells or CD4^+^ cytotoxic T cells. To investigate the efficacy of Alphataxin to elevate CD4^+^ T cell numbers under differing conditions, orthotopic studies were conducted using varying numbers of implanted adenocarcinoma cells, Alphataxin doses, and time of initiation of treatment. Not surprisingly, tumor load, initiation of treatment after implantation, and length of treatment affected treatment efficacy, and this supports the observations that in human cancer, tumor load and early treatment initiation are critical for treatment success (37).

Renal adenocarcinoma is frequently not diagnosed until after metastasis, a disease stage for which there are few effective prophylactic or curative treatment options; however, recent promising advances demonstrate that liquid biopsy of easily accessible blood-based biomarkers provide early detection of renal adenocarcinoma (38). Metastatic renal adenocarcinoma patients with the best prognosis have median overall survival of 43.2 months, and those with the worst prognosis have median overall survival of 7.8 months (39). In studies presented here, the suppression of lung metastasis in mice in response to combination treatment is hypothesized to have resulted from decreased orthotopic tumor growth. This hypothesis is supported by the lack of correlation between metastasis and circulating T cells suggesting that the TILs that suppressed kidney tumor growth were responsible for suppressed metastasis. Thus, with early detection and lower tumor load, early initiation of therapy using Alphataxin in combination with anti-PD-1 or other checkpoint inhibitors may decrease tumor growth leading to suppression of metastasis thereby promoting durable remission. Importantly, other than the findings reported here, there are few, if any, effective treatments to prevent lung metastasis in renal adenocarcinoma.

In human patients receiving α1PI therapy, evidence showed that α1PI regulates maturation of DPs to CD4^+^ T cells, as opposed to DPs defaulting to CD8^+^ T cells, and that circulating T cells exhibit sinusoidal changes (17, 24). The α1PI evidence in humans as well as the Alphataxin evidence presented here in mice suggests Alphataxin also influenced maturation of DPs to CD4^+^ T cells. It was striking that DPs were increased after 21 days of treatment regardless of the treatment arm considering that thymopoiesis in adult mice requires 21 days (35). DPs are mostly found within the thymus as precursors to mature CD4^+^ or CD8^+^ T cells although they are also found in circulation in many species with as yet uncharacterized function (40). Notably, the increase in the number of DPs observed here was significantly greater in the combination treatment arm than in other treatment arms, suggesting that combination treatment markedly influenced thymopoiesis. The reason for increased circulating CD4^+^ T cells and CD4/CD8 ratio in the monotherapy arms, but not in the combination-treatment arm is unclear, but might be related to the increase in the number of CD4^+^ TILs in the combination-treated mice consequently diminishing the number of circulating CD4^+^ T cells and CD4/CD8 ratio.

Alphataxin and α1PI function by stimulating cellular locomotion and endocytosis induced by LDL-RFMs including functionally-related receptors and their cargo (28). The data presented here introduce several new questions. While α1PI and the complex between HLE-CS and α1PI have been previously shown to bind to LDL-RFMs, HLE-CS in complex with Alphataxin induces the same cellular locomotion and endocytosis without the participation of α1PI (15, 41, 42). This raises the fundamental question of the role played by HLE-CS in cellular locomotion and endocytosis. Another question introduced here is how cytokines in blood or tumors interact with T cells in their anti-tumor effects. Cytokine communication between CD4^+^ T cells and CD8^+^ T cells would be expected to be initiated upon endocytosis of pathologic cargo, not physiologic cargo, yet this needs to be investigated. Considering that Alphataxin binds to the active site of HLE-CS in the same manner as α1PI, much can be inferred from clinical trial data using α1PI; however, additional studies on Alphataxin induced CD4^+^ TILs including CD8 depletion studies and examining the effects on Tregs would be informative. The Alphataxin induced elevated presence of CD4^+^ T cells within tumors and consequential enhancement of CTL outcome amends our definition of exhausted CTLs and the mechanisms of their ontogeny (1). Additional studies in similar mouse models would strengthen the conclusions regarding which types of tumors Alphataxin might be useful. Nonetheless, the implications of these findings are that Alphataxin and anti-PD-1 together amplify the drug effects of monotherapy and provide a promising application in renal adenocarcinoma.

Alphataxin is orally available, appears to be nontoxic, is potentially protective, and is effective in suppressing tumor growth, regressing tumors, and preventing metastasis. Alphataxin exhibited no adverse effects; rather, 10 mg/kg Alphataxin showed a potentially protective effect since, as compared with untreated mice, fewer Alphataxin-treated mice in monotherapy arms or the combination arm were moribund requiring early euthanization. The comparison of complete blood cell counts after Alphataxin monotherapy or combination treatment showed no changes in any blood cell types other than an increase in the number of lymphocytes and RBCs suggesting that Alphataxin, like α1PI, did not disrupt circulating blood cell homeostasis. Further investigation is needed to explain how Alphataxin and α1PI treatment induce increased RBCs and whether the effects are on increased erythropoiesis or decreased eryptosis (RBC programmed cell death).

In conclusion, results suggest that oral delivery of Alphataxin has the potential to be a safe and potentially protective treatment in the secondary immunodeficiency of cancers other than renal adenocarcinoma. The data presented are compelling that Alphataxin in combination with an immune checkpoint inhibitor provides a powerful approach that can produce long-term remission in T cell-responsive tumors.

## Data Availability Statement

The raw data supporting the conclusions of this article will be made available by the authors, without undue reservation.

## Ethics Statement

The studies involving human participants were reviewed and approved by Copernicus Group Independent Institutional Review Board, Durham, NC. The patients/participants provided their written informed consent to participate in this study. The animal study was reviewed and approved by Division of Laboratory Animal Resources, Stony Brook University (IACUC# 250725); AJES Life Sciences, Stony Brook, NY (IACUC# 764665-4); Charles River (Morrisville, NC, IACUC# 990301).

## Author Contributions

CB conceived, designed, performed statistical analysis, and wrote the paper. RW conceived and designed the studies. MR performed data analysis. All authors contributed to the article and approved the submitted version.

## Funding

Funding for these studies was generously provided by the Harry Winston Research Foundation to CB.

## Conflict of Interest

CB and RW co-own related patents US 10,413,530 and US 10,709,693.

The remaining author declares that the research was conducted in the absence of any commercial or financial relationships that could be construed as a potential conflict of interest.

## Publisher’s Note

All claims expressed in this article are solely those of the authors and do not necessarily represent those of their affiliated organizations, or those of the publisher, the editors and the reviewers. Any product that may be evaluated in this article, or claim that may be made by its manufacturer, is not guaranteed or endorsed by the publisher.

## Abbreviations

α1PI, alpha-1 antitrypsin, alpha-1 proteinase inhibitor, HLE-CS, cell-surface human leukocyte elastase, HLE-G, granule-associated human leukocyte elastase, VLDLR, very low density lipoprotein receptor.
